# A Novel Optical Scanning Technique with an Inclined Focusing Plane

**DOI:** 10.1038/s41598-019-39415-8

**Published:** 2019-02-27

**Authors:** Andrey Alexandrov, Giovanni De Lellis, Valeri Tioukov

**Affiliations:** 1 I.N.F.N. sezione di Napoli, I-80126 Napoli, Italy; 2 Università degli Studi di Napoli Federico II, I-80126 Napoli, Italy; 3 National University of Science and Technology MISIS, RUS-119049 Moscow, Russia; 4 Lebedev Physical Institute of the Russian Academy of Sciences, RUS-119991 Moscow, Russia

## Abstract

We propose a novel technique for fully automated optical scanning of thin samples. We analyze its performance and estimate the achievable scanning speed to compare it with conventional techniques. It paves the way to the next generation of highspeed scalable scanning systems, at least one order of magnitude faster than existing ones. We show that the efficiency and the accuracy of this new technique are comparable to those of the conventional ones, while the scanning speed scales proportionally with the number of cameras installed, hence the large expected improvement.

## Introduction

The success of the DONUT^1^ and CHORUS^2,3^ experiments has marked the revival of the nuclear emulsion technique for charged particle detection. Recently, the OPERA^4,5^ experiment has brought it forward demonstrating the feasibility of multi-ton scale detectors with millions of emulsion films. It was thanks to the development of fully automated optical microscopes that the analysis of large emulsion volumes became possible. The Emulsion Cloud Chamber (ECC) technique^6^, extensively used by the OPERA collaboration, has made thin (about 50 *μm*) double-sided emulsion films^7^ to become a standard, used nowadays in most of emulsion applications in fields like neutrino physics, directional Dark Matter (DM) search, nuclear physics, medical applications and muon radiography for geology and archeology. An especially challenging demand on high-speed scanning systems comes from the DM search, where, for the sake of competitiveness with other experiments, tons of emulsion films will ultimately have to be analyzed with unprecedented accuracy on the time scale of a few years. Technological progress allows creating scalable multi-camera setups with huge image data flow processed in real time with the help of multiple powerful Graphical Processing Unit (GPU) boards. On the contrary, the mechanical part of the microscope cannot be replicated to get a proportional gain in the scanning speed, thus, making the sample movement a bottleneck for the overall scanning system performance, especially for the analysis of thin emulsion films. Moreover, as it will be shown below, the replacement of mechanical stages with more performant piezo actuators gives only a limited effect. Therefore, the optimization of the scanning process with the technique proposed here is important to overcome the performance limitations. We report a simple and cost-effective technique for fast optical scanning of thin samples.

## State of The Art

**Figure 1:**
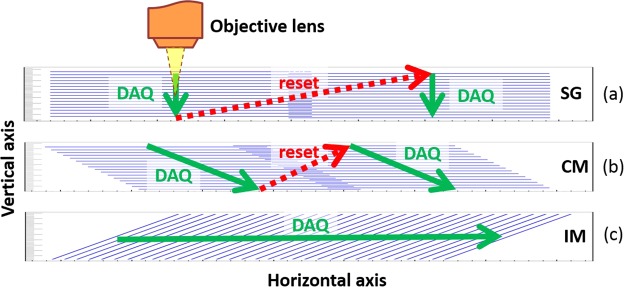
Illustration of (**a**) Stop&Go (SG), (**b**) Continuous Motion (CM) and (**c**) the proposed Inclined Motion (IM) scanning techniques.

An automatic digitizing of the 3D optical content of nuclear emulsion films is realized by moving the focal plane of the objective lens inside the sample and taking a series of tomographic images while in motion. The simplest and the oldest motion method, used already in the first automated microscopes, is the so-called Stop&Go (SG) technique. It is illustrated in Fig. 1a and includes two steps called the data acquisition (DAQ) motion and the reset motion. In case of the SG, the DAQ motion involves only vertical movement of the objective lens and, therefore, depends only on the camera frame rate and the desired sampling step (the distance along the Z-axis between two consecutive frames). The reset motion, in turn, is intended to prepare the readout of the next field of view (FoV) by moving the sample to the required position, and, therefore, it can involve movement along all possible axes. Since the horizontal dimensions of the microscope’s FoV are usually much larger than sample’s thickness, the SG performance is limited by the horizontal motion of the sample. The SG provides a vertical stack of tomographic images: the most convenient format for processing.

The Continuous Motion (CM)^8,9^ is a more recent technique, illustrated in Fig. 1b. In this approach the objective lens (or the sample) performs periodic oscillations along the vertical axis while moving at a constant speed in the horizontal plane. The movements are synchronized such that during one period of the objective lens vertical oscillation the horizontal stage displaces exactly one FoV. Therefore, unlike the SG, it produces a tilted set of tomographic images. The CM technique provides a significant boost in the scanning speed for thin samples with respect to the SG since the CM reset motion time is not limited by the horizontal motion but only by the performance of the vertical stage.

Both the CM and the SG techniques involve the reset motion. In addition, some time is spent to speed up and slow down objective lens or sample movements. The time fraction, when no useful images are grabbed, represents the “deadtime” of a microscope data taking and can vary with the implementation and hardware. To give an idea, for a system described in ref.^10^ the reset time fraction amounts to 70% of the total time, while application of the CM reduces it to 34%, still a noticeable fraction. A new scanning technique with zero reset time would noticeably increase the scanning speed by fully exploiting the microscope hardware.

## The Inclined Motion Technique

**Figure 2:**
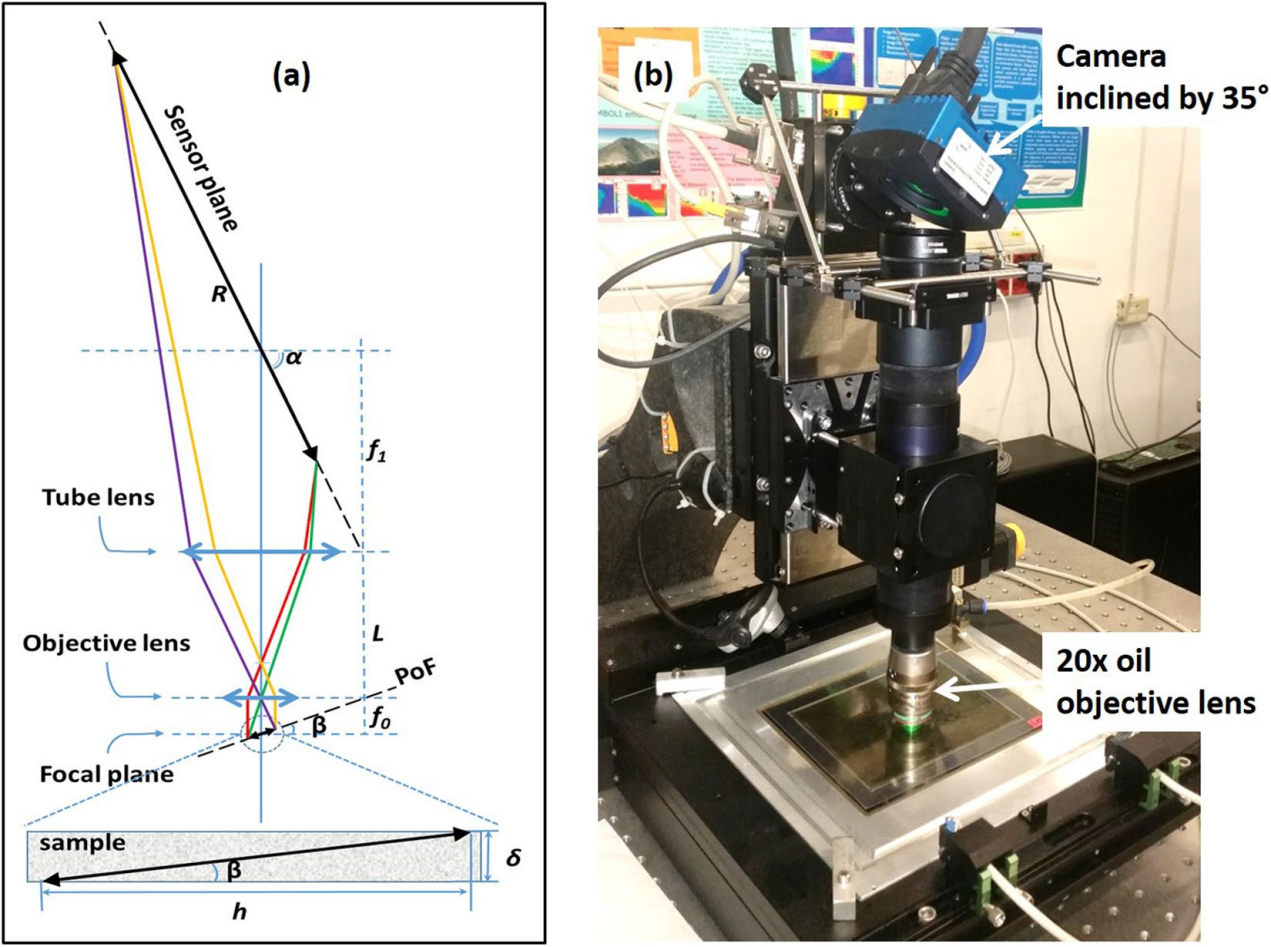
(**a**) Image forming paths in a microscope with a sensor plane inclined by an angle *α*. (**b**) Picture of the microscope with an inclined camera used for tests.

The in-depth scanning of the sample volume can be accomplished not only by taking piles of horizontal images, like the SG and the CM do. If the sample is thin and the FoV is wide enough it becomes possible to incline the focal plane (FP) of the objective lens such that it would span across the depth of the volume. Then, as shown in Fig. 1c, displacing horizontally the inclined FP, one can digitize the whole volume of the sample. Normally, image (film or sensor) planes of a microscope and the FP of the objective lens are parallel to each other and perpendicular to the optical axis. Therefore, the plane of best focus (PBF) coincides with the FP. If one rotates the camera, the inclination of the sensor plane will lead to the inclination of the PBF. Figure 2a shows image forming light trajectories inside a microscope with an inclined camera. The relation between inclination angles of the PBF (*β*) and the image sensor plane (*α*) is given by the following equation:

1 $$\tan (\beta )=\frac{n}{M}\,\tan (\alpha ),$$

where *M* = *f*_1_/*f*_0_ is the magnification of the objective lens and *n* is the refraction index of the immersion media. The FoV depth (*δ*) and the length (*h*) along the inclined axis can be calculated using:

2 $$h=\frac{R}{M}\,\cos (\alpha ),$$

3 $$\delta =n\frac{R}{{M}^{2}}\,\sin (\alpha ),$$

where *R* is the camera sensor width. Thus, the maximal scan depth depends only on the sensor size and the magnification of the objective lens.

## Results

In order to perform the proof-of-principle test we assembled a setup with a possibility to incline the camera as shown in Fig. 2b. We used an infinitely-corrected 20×, 0.75 numerical aperture (NA) and 350 *μ*m working distance (WD) Nikon multi-immersion objective lens (*f*_0_ = 1 *cm*), a standard (*f*_1_ = 20 *cm*) Nikon tube lens and a Mikrotron MC-4082 camera with the effective sensor width R = 14.7 *mm*. The bright-field illumination was a standard Kohler type. An immersion oil with *n* = 1.515 was used as an immersion media. The magnification was not constant across the FoV, ranging from *M* = 20.04 at one edge to *M* = 20.42 at the opposite one. The magnification in the FoV center was measured to be 20.16 ± 0.02.

**Figure 3:**
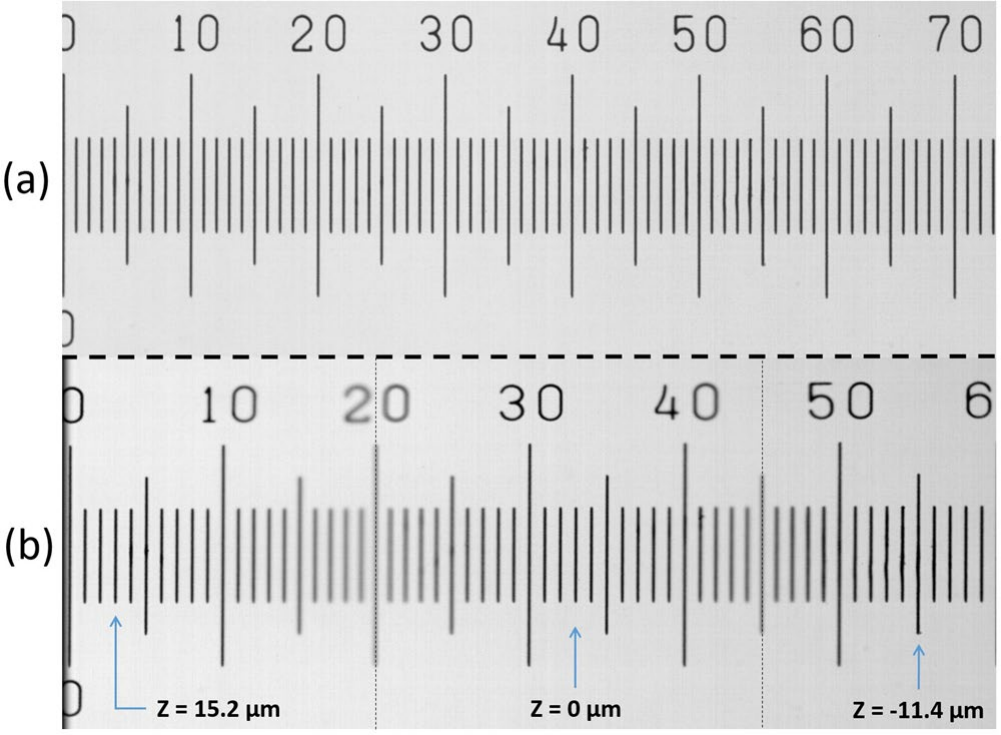
(**a**) A micrometric ruler image obtained with a camera normal to the optical axis. (**b**) The same micrometric ruler seen with an inclined camera. The image is composed of 3 frames taken at different depths. Arrows show the most focused ticks at each frame and their measured depths. The distance between short vertical lines is 10 *μ*m.

Figure 3a shows a microscopic ruler observed at microscope with the camera perpendicular to the optical axis. The ruler is focused throughout the entire length, since it is parallel to the PBF. After the camera rotation around the Y axis by 35°, only the central part remains in focus. Figure 3b is composed of 3 frames taken at different depths corresponding to the focused position of both edges and the central part connected at ticks 20 and 45. Arrows in Fig. 3b show the most focused tick for each frame and the corresponding Z coordinate of the frame. The FoV width is about 600 *μ*m, in agreement with the value predicted by the formula (2): *h* = 597 ± 5 *μm*. An estimate of the FoV inclination angle is performed using coordinates of the leftmost and the rightmost focused lines in Fig. 3b (marked with arrows) and gives *β* = 2.9° ± 0.2° that is in agreement with the value predicted by the formula (1): *β* = 3.0° ± 0.4°. The varying magnification along the inclined horizontal axis can be noticed from the difference in observed distances between ticks 1 and 30, and ticks 30 and 59. After the camera inclination, the difference of optical magnification at the opposite edges is small (≈2%). The observable horizontal magnification increase in Fig. 3b (≈20% with respect to Fig. 3a) is a pure geometrical effect: the effective cross-section of the rotated sensor reduces as cos(*α*) and a smaller image fraction can be readout, but since the number of pixels remains the same, in a digitized image one sees it as a magnification increase along the inclination axis.

For the sample with the thickness of about 30 *μ*m, the minimal camera inclination angle in this case can be calculated with the formula (3). Substituting *δ* = 30 *μm*, *R* = 14.7 *mm*, *M* = 20.16 and *n* = 1.515 one gets *α*_*min*_ ≈ 33°. We have chosen to set it to *α* = 35° in order to have some margins below and above the sample to compensate its local curvature.

**Figure 4:**
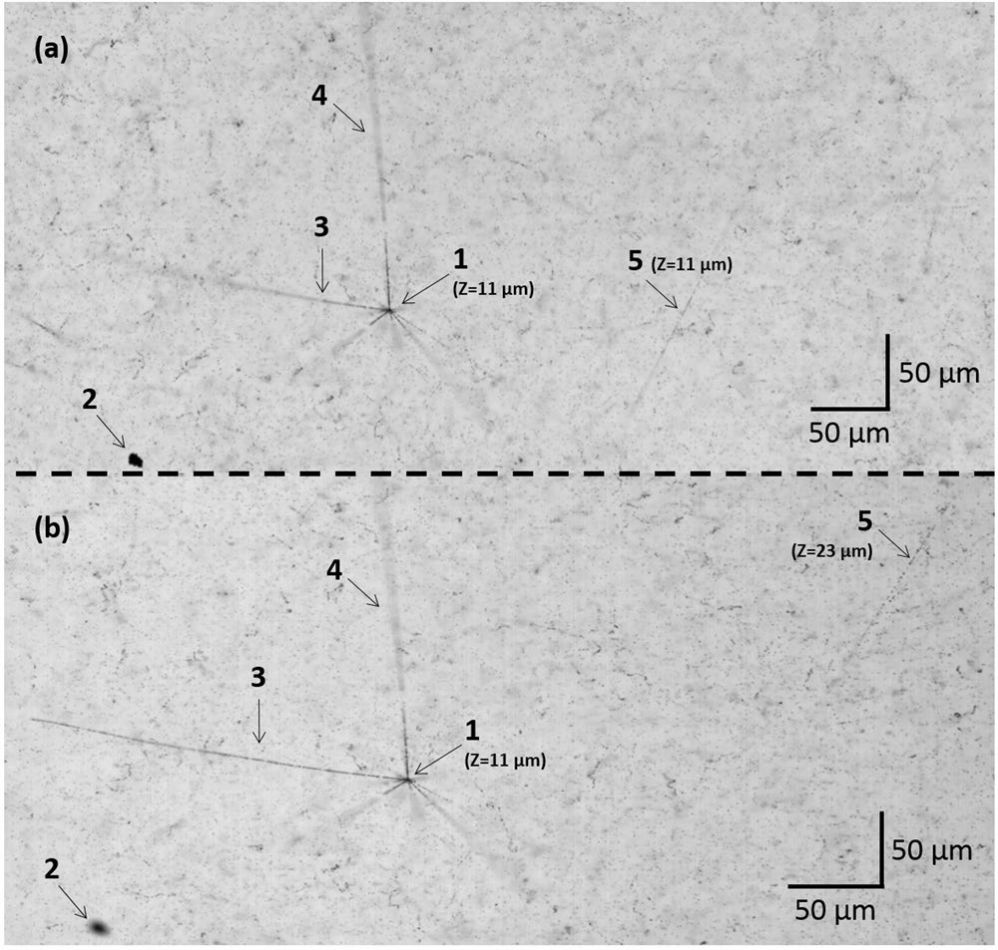
Nuclear emulsion sample images taken with non-inclined (**a**) and inclined (**b**) cameras. Both images have the decay vertex 1 focused. The black spot 2 is out of focus in the inclined image (**b**). The decay track 3 has the same inclination angle as the PBF in (**b**) and, therefore, stays focused. The decay track 4 is heading in the direction that is almost perpendicular to the inclination one and, therefore, it looks the same in both images. The cosmic ray 5 appears to be at different positions in the images.

The test was performed using an OPERA-like emulsion film exposed to a 6 GeV/c *π*^−^ beam at CERN. A charged particle passing through emulsion activates silver halide crystals along its path. During the emulsion film development activated crystals are transformed into grains of metallic silver with diameters of several hundreds nanometers visible in microscope as tiny dark spots. Figure 4a shows an example of the emulsion content seen in an optical microscope. Low ionizing pions, directed horizontally (along the X axis in the image), produce rather sparse tracks that are barely visible with a naked eye in a “sea” of spurious grains (so-called fog) produced by thermal activation of silver halide crystals in emulsion. Higher ionizing particles, produced in nuclear interactions of pions, leave tracks with higher grain density emerging from a common vertex (vertex 1 and tracks 3 and 4). As soon as the emulsion film is produced, it starts to accumulate cosmic ray tracks. During the storage and transportation the film was kept vertically and, therefore, the majority of cosmic ray tracks are directed more or less along the Y axis like the track 5, being almost perpendicular to the beam of pions.

Figure 4 shows a comparison between images taken in the same place inside the emulsion with non-inclined (4a) and inclined (4b) camera setup. The image quality is indistinguishable by visual inspection. The image shows a decay vertex 1 with a number of tracks emerging from it. Two of them, tracks 3 and 4, are heading in direction almost coincident with *X* and *Y* axes, respectively. The decay vertex 1 is in focus in both images at *Z* = 11 *μm*. The black spot 2, that has the same *Z* as the vertex 1, is out of focus in the latter image due to the inclination of the PBF along the *X*-axis. The decay track 3, heading in negative *X* and negative *Z* directions, becomes rapidly unfocused in Fig. 4a. Due to its slope occasionally equals to the PBF inclination angle, the track 3 stays focused in Fig. 4b until it leaves the emulsion volume. On the contrary, since the track 4 emerges perpendicularly to the inclination direction, it looks almost the same in both images. Inclined and non-inclined PBFs intersect the large-angle cosmic ray track 5 at different depths, making it appear at different positions within images with respect to the vertex 1.

We scanned the same sample volume with two methods: the conventional SG and the IM, reported in this paper. In order to estimate the repeatability of the measurements and the intrinsic accuracy of the analysis method in use, the SG method was applied twice. We applied the same image processing procedure for all datasets. The local frame coordinates for the IM dataset were recalculated to take into account the inclination of the PBF using the formulas:

4 $$x=iP(i)\,\cos (\alpha );\,\,y=jP(i);\,\,z=iP(i)\frac{n}{M}\,\sin (\alpha ),$$

where *x*, *y*, *z* are expressed in *μ*m and *i*, *j* are image coordinates in pixels with respect to the image center. The *P* function represents the varying pixel-to-micron conversion factor accounting for the different magnification along the inclination axis. Its calculation is described in details in the Methods section.

**Figure 5:**
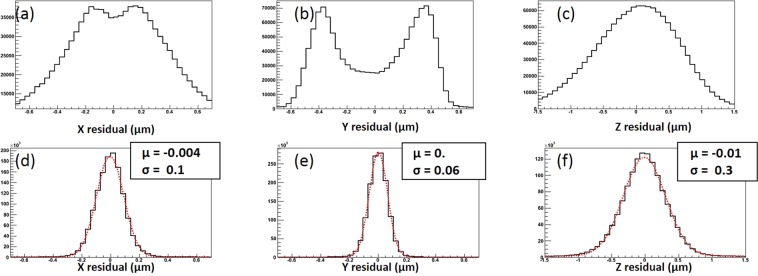
(**a**–**c**) Grain matching residuals along X, Y and Z axes respectively before applying the correction matrix. (**d**–**f**) Same residuals with the corrections applied.

We applied the standard grain reconstruction procedure^11^ to both datasets. Despite the same coordinate system introduced by the formulas (4), the different magnification and optical distortions due to the inclination of the PBF lead to a discrepancy between the real grain coordinate and the reconstructed one, worsening the spatial accuracy. This effect is clearly visible in the Fig. 5a–c, where residuals between matched grain from SG and IM datasets have a wide double-peak structure. Full analytical expression that would take into considerations all possible distortion effects would be rather difficult to derive and would include a large number of parameters known with limited precision. The approach we followed is more practical and uses simple analytical expressions (4) to make the first order approximation taking into account only most prominent effects. Then we used the SG dataset as a reference sample and introduced a special correction matrix to cope with second order effects. Given the larger statistics, the correction matrix can be generated to any degree of precision. The generation procedure is described later in the Methods section. Figure 5d–f show residuals of the grain matching procedure between the IM and SG datasets after application of the correction matrix. The correction matrix significantly improves the residuals and the dual-peak structure disappears. The accuracy for the Y coordinate (Fig. 5e) is better than that for X since its calculation does not involve the *α* angle that is known with a finite precision.

**Figure 6:**
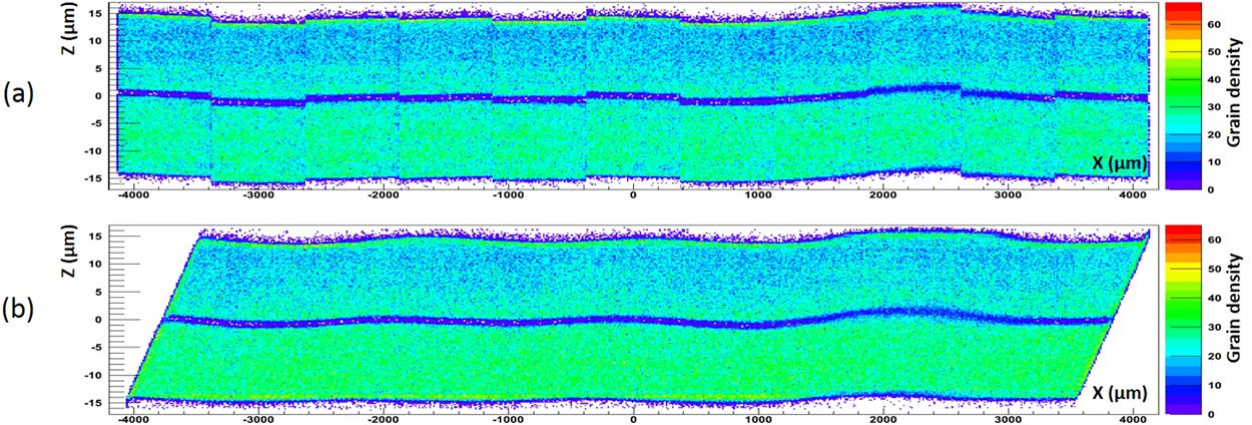
The XZ profile of reconstructed grains (**a**) with the SG and (**b**) with the IM methods. The (online) color scale is the grain density.

Figure 6a,b show the XZ profile of reconstructed grains in the SG and the IM datasets respectively. The color scale indicates the local grain density. The low density (violet-blue) line in the middle of the emulsion film is a 1-*μ*m-thick insensitive layer of pure gelatin formed during film production. Some excess of grain density near surfaces and the depth density gradient are due to imperfect treatment during the film development. The curved section between 2000 and 3000 *μ*m is due to a small bubble entered between the emulsion film and the glass support. The blurring of the middle line indicates that this section is curved also along the Y axis. The discrete view structure in the Fig. 6a is typical of the SG method and is due to strong vibrations during the vertical DAQ motion produced by heavy and unbalanced optical group driven by the vertical axis. The misalignment of views is usually corrected by allowing some overlap between neighboring views and matching grain patterns therein. On the contrary, the IM technique shows better intrinsic alignment of the reconstructed data since its horizontal DAQ motion involves only the movement of a light, balanced and rigid frame with a sample on it, producing much less vibrations.

**Figure 7:**
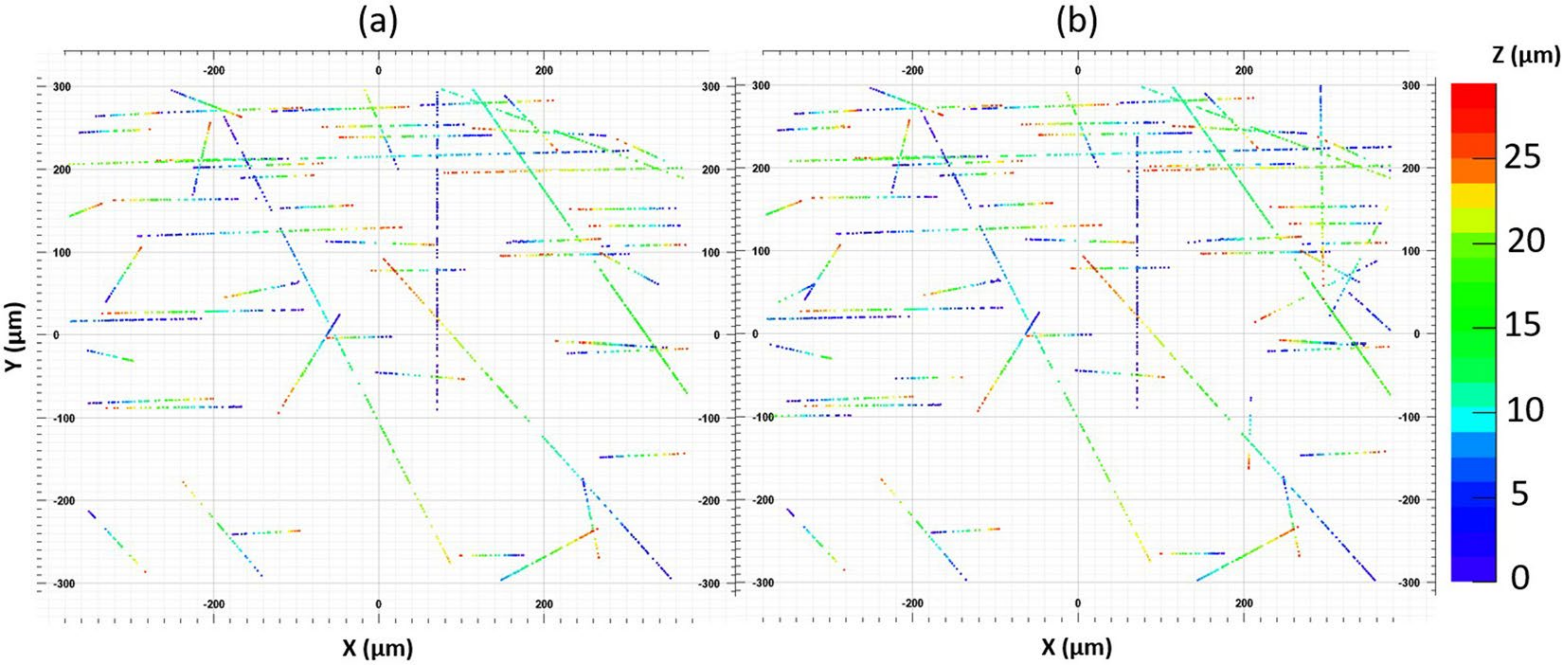
Reconstructed microtracks of the same field of view (**a**) in the SG and (**b**) in the IM datasets.

Finally, we applied the same microtrack reconstruction procedure to grains in both datasets. The reconstructed microtracks of the same FoV for both the SG and IM datasets are shown in Fig. 7a,b respectively. Microtracks parallel to the X axis are produced by pions from the beam, while other microtracks are due to cosmic rays or nuclear decay products.

For the performance analysis, we considered only microtracks with angles larger than 1.25 rad and longer than 90 *μ*m. This selection allows controlling the quality of the introduced correction procedure since a minimal uncorrected distortion would make these long large angle microtracks appear curved. This would in turn deteriorate the reconstruction efficiency, since the microtracking procedure is optimized to reconstruct straight tracks. Long microtracks are formed by a sufficiently large number of grains, at least 17, to completely eliminate the combinatorial background due to random coincidences of several grains. This selection allows evaluating the uncertainty introduced by this new scanning approach on the measurement of microtracks. The analyzed sample volume consists of 97 views of the SG mode, equivalent to 43 *mm*^2^. It was scanned with both SG and IM techniques. Every microtrack reconstructed in either dataset was confirmed by visual inspection. SG and IM datasets contained 1900 and 1968 microtracks respectively, with 1871 microtracks reconstructed in both datasets. The combined dataset contained 1997 microtracks. Hence, the coincidence level (*CL* = |*SG* ∩ *CM*|/|*SG*∪*CM*|) is 93.7%, with the IM showing even a little bit higher efficiency than the SG: 98.5% against 95.1%, respectively. A slightly better efficiency in the IM case can be explained by stronger vibrations during vertical DAQ motion for the SG. On the contrary, the horizontal DAQ motion, as in the IM case, involves only a light, balanced and rigid frame with a sample on it, causing much less vibrations. Since the correction matrix calculation involves tens of SG views, vibrations are averaged out and, once the matrix is applied, reconstructed grains in the IM dataset turn out to be less distorted from their true positions than in the SG one.

**Figure 8:**
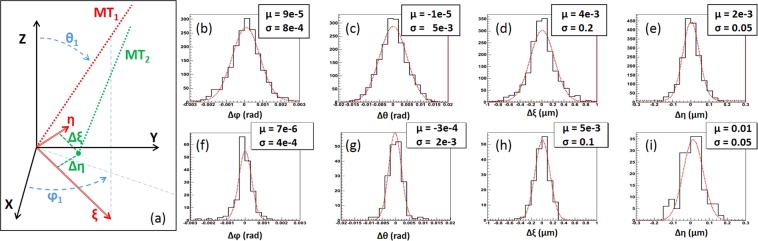
Inset (**a**), *ϕ* is the angle between the *X* axis and the microtrack (MT _1_) projection on the *XY* plane. *θ* is microtrack angle with the *Z* axis. *ξ* axis is perpendicular to the microtrack vector and lies in the plane formed by microtrack’s vector and the *Z* axis. *η* axis is perpendicular to microtrack vector and the *ξ* axis. (**b**) to (**e**) SG-IM matching residual plots. (**f**) to (**i**) SG-SG matching residual plots.

The SG-IM matching accuracy was estimated by calculating residuals between matched microtracks. The calculation procedure is shown in Fig. 8a: *ϕ*_1_ is the angle between the *X* axis and the SG microtrack (*MT*_1_) projection on the *XY* plane. *θ*_1_ is *MT*_1_ angle with the *Z* axis. *ξ* axis is perpendicular to the *MT*_1_ vector and lies in the plane formed by *MT*_1_ vector and the *Z* axis. *η* axis is perpendicular to both *MT*_1_ vector and the *ξ* axis. Then, the Δ*ξ* and Δ*η* residuals of a matched IM microtrack (*MT*_2_) can be calculated as the distance between central points of *MT*_1_ and *MT*_2_ in the *ξη* coordinate system orthogonal to the *MT*_1_. The choice of Δ*ξ* and Δ*η* residuals allows inclusion of *Z*-coordinate measurement error only in Δ*ξ* residual, while Δ*η* residual depends only on *XY* errors. The SG-IM matching residuals are shown in Fig. 8b–e.

In order to understand the accuracy of the measurement method, we rescanned a reduced sample volume equivalent to 9 FoVs with the SG technique. The same reconstruction and analysis procedure were applied and the residuals, shown in Fig. 8f–i, were calculated. As expected, residuals in SG-SG case are lower than for the SG-IM, the difference being due to the application of different scanning techniques. The equality of Δ*η* residuals (Fig. 8d,i), means that all the difference comes from the uncertainty of the grain Z-coordinate reconstruction. Indeed, the longitudinal optical resolution is several times worse than the traversal one and this fact also is also visible in the Z residuals (Fig. 5f), 3 times larger than X residuals (Fig. 5d).

Once the IM technique replaces the SG one, *ϕ* and *θ* of reconstructed microtracks will stay within 0.7 and 4.6 mrad from their previous values, respectively, while the coordinate difference will not exceed 200 nm. The differences, introduced by the IM method, are small and reside within the tolerances (for large-angle tracks, typically, 10 and 20 mrad for *ϕ* and *θ*, respectively) required for the next steps of data analysis.

## Discussion

Horizontal movement of the inclined PBF sweeps a volume corresponding to a depth equal to *δ*. As shown in Fig. 1c, data acquisition during this motion produces a horizontal pile of inclined images. The scanning speed can be calculated using the following formula:

5 $${v}_{IM}=w\frac{sf}{\tan (\beta )}=w\frac{sfM}{n\,\tan (\alpha )},$$

where *w* is the FoV dimension in the direction perpendicular to the movement, *s* is the desired step along the Z-axis (sampling step) and f is the camera frame rate. The camera inclination angle for the desired *δ* can be obtained using equation (3). In case of *N*_*cam*_ cameras the equation for *α* can be rewritten to take them into account:

6 $$\alpha =\arcsin (\frac{\delta {M}^{2}}{nR{N}_{cam}}).$$

In our setup $({M}^{2}\delta )/(nR)\, \sim \,0.55$, therefore, already with 2 cameras the *α* angle becomes small and one can rewrite formula (5) as:

7 $${v}_{IM}\approx w\frac{sfR}{\delta M}{N}_{cam}.$$

Thus, the scanning speed of a multi-camera system operating in the IM mode is proportional to the number of cameras.

**Figure 9:**
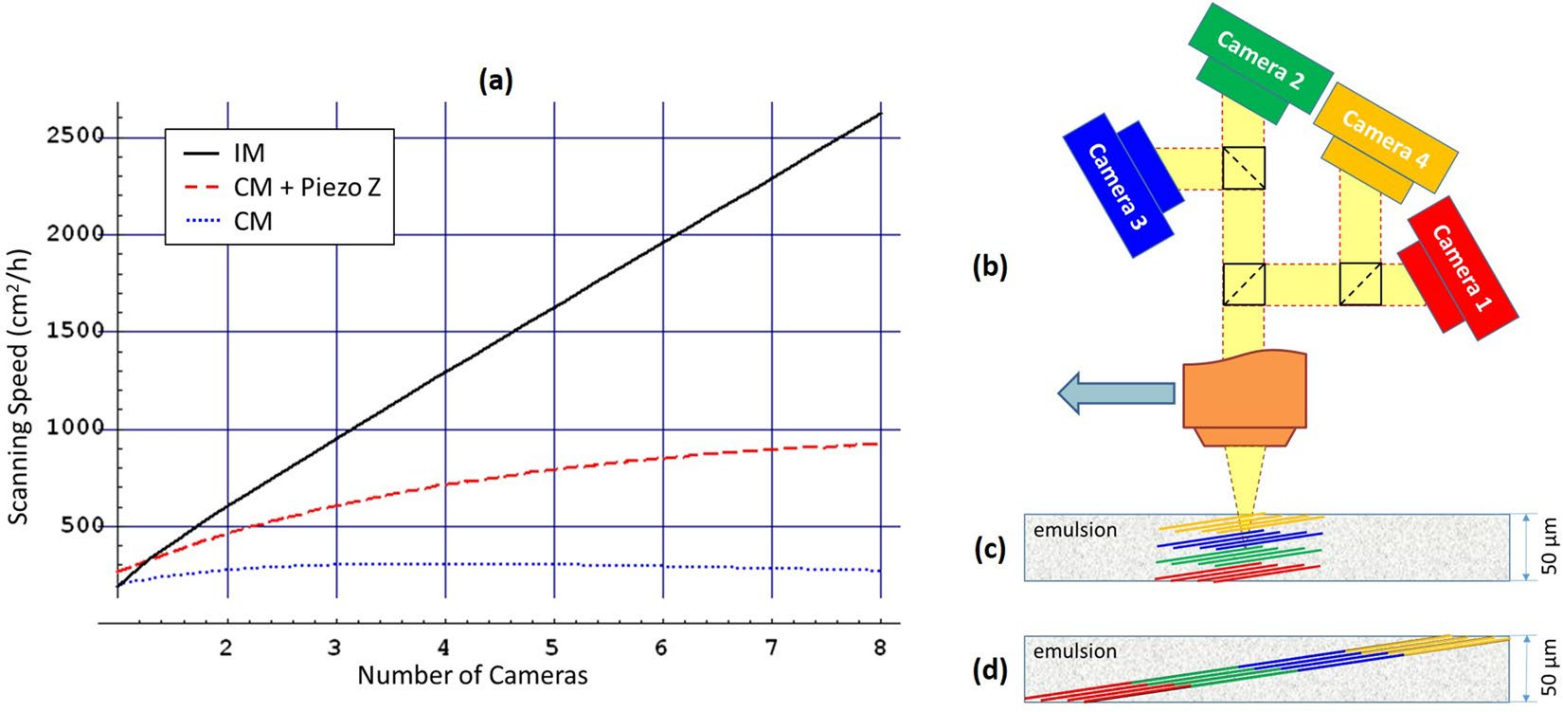
(**a**) Scanning speed dependency on the number of cameras used. Calculations are performed for 4 M cameras running at 563 fps and 50 *μ*m thick emulsion films. (**b**) A possible configuration of a multi-camera system that performs in-depth splitting of the FoV. (**c**) PBFs configuration in case of in-depth splitting. (**d**) PBFs configuration in case of FoV splitting.

Figure 9a shows the estimated scanning speed comparison of a multi-camera scanning system between the IM (black solid line), the CM (blue dotted line) and the CM with a piezo-accelerated Z stage (red dashed line). For systems with several cameras the scanning speed of the IM is directly proportional to the number of cameras, while the CM shows an asymptotic behavior. Indeed, in the CM, increasing the number of cameras the working cycle starts to be more and more dominated by the reset phase and the scanning speed increase vanishes. For the system with a piezo-accelerated Z stage the reset phase is much shorter and, therefore, the maximal achievable scanning speed is higher than that of the system equipped with a standard Z stage. Anyway, the piezo-accelerated Z stage has only a limited effect giving unproportionally little gain with increasing number of cameras. Absence of the reset motion phase in the IM technique keeps the scanning speed proportional to the number of cameras and makes it perfectly adapted for multi-camera scanning systems.

From equation (7) it is possible to figure out several approaches for implementation of multi-camera systems. The first one is to focus each camera at different depths inside the emulsion bulk thus reducing the effective scanning volume thickness *δ*′ = *δ*/*N*_*cam*_ swept by each camera. An example of such a multi-camera setup is shown in Fig. 9b, where the imaging beam is split into four identical full-view-sized beams by means of beam splitters. Placing cameras at different distances from the objective lens results in a set of PBFs displaced in-depth along the optical axis as shown in Fig. 9c. This configuration does not require a wide-field optics and is quite flexible allowing adjustment to different emulsion film thicknesses by varying PBF angles and distances between them. The second option is to create a longer array of sensors with *R*^′^ = *R* × *N*_*cam*_ either by putting them close together or by splitting the imaging beam in the way that every camera observes only a part of the FoV resulting in the set of PBFs as shown in Fig. 9d. This option requires a wide-field objective lens limiting its practical application. The third option is to split the imaging beam in the way that every camera produces equal full-view-sized image at the same depth and trigger the cameras to take pictures one at a time in a consecutive manner thus creating a virtual camera with an effective frame rate *f* ^′^ = *f* × *N*_*cam*_. This option does not require a wide-field optics but, unlike the first one, its application is limited to thin emulsion films only since the maximal scanning depth is equal to that of a single camera.

Another important advantage of the proposed IM technique implementation is that it is applicable to all objective types. Indeed, an alternative solution would be an inclination of the entire optical group, including also the illumination system. But that solution would be applicable to dry objective lenses only, since it would require working distances larger than 500 *μm*. On the contrary, the IM technique implemented by means of the camera rotation, as proposed in this paper, overcomes this limitation and can be used with immersion objective lenses of any working distance.

The maximal scanning power of a laboratory is limited by both the available budget and room space to host microscopes. The cost of a single camera and a frame-grabber is usually much cheaper than that of the entire microscope. Therefore, the multiplication of cameras offers a simple and cost-effective way of boosting the scanning power of both a single microscope and a scanning laboratory as a whole.

The development and implementation of the proposed IM technique allows boosting of readout speeds by more than one order of magnitude, thus enabling the achievement of much more challenging goals. For example, one order of magnitude (2000 cm^2^/h) with respect to the current NGSS speed (190 cm^2^/h)^9^ is achievable already with 6 cameras. The maximal achievable scanning speed with the IM technique can be limited by the hardware in use. If we consider the NGSS microscope then the first limit comes from the camera. With the minimal exposure time of 2 *μ*s and the pixel size of 0.35 *μ*m, the stage displacement with a speed faster than 17.5 mm/s will cause blurring, limiting the scanning speed to 3000 cm^2^/h. This limit can be overcome by introducing a stroboscopic illumination with light pulses shorter than 2 *μ*s. Another potential limit on the scanning speed is due to the horizontal stage performance. Indeed, the maximal speed and acceleration rate of the NGSS stage would limit its scanning speed to about 6000 cm^2^/h. This limit can potentially be extended to about 40000 cm^2^/h, if the stage is upgraded to the fastest commercially available one.

The application of the IM technique for readout of nuclear emulsion films can be particularly fruiful in the fields of high energy, astroparticle, nuclear and medical physics. To name a few particular applications:

- The FOOT^12,13^ (Fragmentation Of Target) experiment is designed to study the interactions of carbon ion and proton beams in the patient tissues in order to optimize the hadron-therapy treatment planning systems. It will also conduct studies with ions of interest for the radioprotection in space missions. The emulsion spectrometer of the detector will use of nuclear emulsions to detect light fragments emitted at large angles;
- The NEWSdm^14,15^ (Nuclear Emulsions for WIMP Search with directional measurement) is the only experiment that uses a solid-state target for directional dark matter search. It uses an innovative approach of detecting the nuclear recoil direction with a nano-emulsion target with the mass of the order of to several tons;
- The neutrino detector of the SHiP^16^ (Search for Hidden Particles) experiment will use a large amount of nuclear emulsion as a tracking media to study tau neutrino physics and search for light dark matter produced in interactions of 400 GeV protons;
- The muon radiography/tomography^17–19^ intended to study the inner structure of volcanoes, geological faults and archaeological sites, where energy-independent and compact emulsion films, unlike electronic detectors, can be easily installed and large instrumented surfaces are required;
- Medical application for hadron-therapy and fragmentation studies^20–23^. The low scanning speed was the main limiting factor for their massive application in this field where a large statistics is needed for precise measurements.
- The IM technique can also be used with samples different from emulsion films, e.g. biological samples, where large volumes have to be analyzed with optical microscopes in the shortest possible time.

## Methods

### Microscope setup

Mechanical components and the illumination system are identical to those described in ref.^10^. The custom imaging system, shown in Fig. 2b, uses same objective lens, tube lens and video camera. In addition, it is equipped with a manual rotation stage that allows rotation of the video-camera around the Y axis and its setting to the desired position.

### Image processing

The image processing consists of the following steps: background image subtraction, signal enhancement by convolution filtering, threshold equalization, clusters reconstruction, correction of optical distortions and compensation of stage vibrations. The details can be found in refs^24,25^ and the references therein.

### Pixel coordinates calculation

As it can be noticed, the image of the micrometer ruler shown in the Fig. 3b is asymmetric, meaning that the magnification is not constant across the inclined PBF. We model the magnification with a second order polynomial function. Its parameters can be calculated by taking measurements of the pixel size (the ratio of the measured FoV dimension over the number of pixels in the sensor along the measurement direction) in three points: near left (*p*_0_, *i*_0_) and right (*p*_2_, *i*_2_) FoV edges and in its center (*p*_1_, *i*_1_). Then, the variation of the pixel size can be written as *P*(*i*) = *ai*^2^ + *bi* + *c*, where *i* is the pixel offset from the center along the X axis. Pixel coordinates relative to the image center are calculated using the formula (4), where the coefficients *a*, *b*, and *c* are defined as:

$$\begin{array}{rcl}a & = & [{p}_{2}({i}_{0}-{i}_{1})+{p}_{1}({i}_{2}-{i}_{0})+{p}_{0}({i}_{1}-{i}_{2})]/d;\\ b & = & [{i}_{2}^{2}({p}_{0}-{p}_{1})+{i}_{1}^{2}({p}_{2}-{p}_{0})+{i}_{0}^{2}({p}_{1}-{p}_{2})]/d;\\ c & = & [{i}_{2}^{2}({p}_{1}{i}_{0}-{p}_{1}{i}_{1})+{i}_{1}^{2}({p}_{0}{i}_{2}-{p}_{2}{i}_{0})+{i}_{0}^{2}({p}_{2}{i}_{1}-{p}_{1}{i}_{2})]/d;\\ d & = & ({i}_{0}-{i}_{1})({i}_{0}-{i}_{2})({i}_{1}-{i}_{2}).\end{array}$$

### Grains reconstruction

The reconstruction algorithm searches for chains of clusters that belong to different consecutive frames and have close X and Y coordinates. Then the found chains are analyzed and patterns corresponding to single grains are isolated. The procedure is described in ref.^11^.

### Correction matrix generation

The matrix is a bi-dimensional array of 3-dimensional offsets to be added to coordinates of every reconstructed grain. To generate it, the same sample volume is scanned with two methods: the SG and the IM. Corrections for the SG are applied and, therefore, this dataset is considered as a reference. Grains from each dataset are matched against each other. Found 3-dimensional offsets between matching grains are filled into a bi-dimensional histogram into a bin with (i, j) coordinates, calculated from known grain coordinates using the inverse (4).

### Microtracks reconstruction

A microtrack of a charged particle in the emulsion sensitive layer is represented by a sequence of grains with the linear grain density along it being higher than that measured in any arbitrary position or direction. The reconstruction procedure uses a 4D-histogram and it is optimized for the reconstruction of straight tracks as described in refs^11,25^.

## Data Availability

The datasets generated and analysed during the current study are available from the corresponding author on reasonable request.
